# Supplementary material for: Ketone body β-hydroxybutyrate restores neuronal Tau proteostasis via ketolysis-independent mechanism
Source: bioRxiv. 2026 Feb 2:2026.01.30.702936. Preprint. [Version 1] doi: 10.64898/2026.01.30.702936 (PMC12889508; doi:10.64898/2026.01.30.702936)

bioRxiv preprint doi: https://doi.org/10.64898/2026.01.30.702936; this version posted February 2, 2026. The copyright holder for this preprint (which was not certified by peer review) is the author/funder, who has granted bioRxiv a license to display the preprint in perpetuity. It is made available under aCC-BY 4.0 International license.

SUPPLEMENTAL FIGURES

Figure S1, related to Figure 1: Ketone ester diet induces mild ketosis, and any weight or food intake differences from the diet are genotype-dependent.

(A) Plasma *R*-βHB levels across Zeitgeber times (ZT) 6-24 for 12 week-old WT mice (C57B/6J) on the KE or CTL diets. AUC, area under the curve.
(B) Plasma *R*-βHB levels at ZT18 at 12 weeks-old as in (A), showing individual mice.
(C) Plasma *R*-βHB levels at ZT18 for 9.5 month-old (mo) CTL and KE diet fed mice.
(D) Initial body weights of WT and hTau+ mice before administration of either diet, illustrating no baseline differences between the groups.
(E) Ending body weights of WT and hTau+ mice at the end of the 16-week KE diet study.
(**F)** Weight change compared to baseline by week for ketone ester diet study.
(G-I) Average weekly food intake (G), average weekly kCals intake (H), and kCals intake by week (I). hTau+ mice consumed more chow regardless of diet.
Data are represented as mean ± SD. * $p < 0.05$, ** $p < 0.01$, *** $p < 0.001$, **** $p < 0.0001$, ns not significant by Šídák's multiple comparisons test (A, B, D, E, G, and H), Welch's t-test (C), and AUC analysis (A, F, and I).

bioRxiv preprint doi: https://doi.org/10.64898/2026.01.30.702936; this version posted February 2, 2026. The copyright holder for this preprint (which was not certified by peer review) is the author/funder, who has granted bioRxiv a license to display the preprint in perpetuity. It is made available under aCC-BY 4.0 International license.

Figure S1

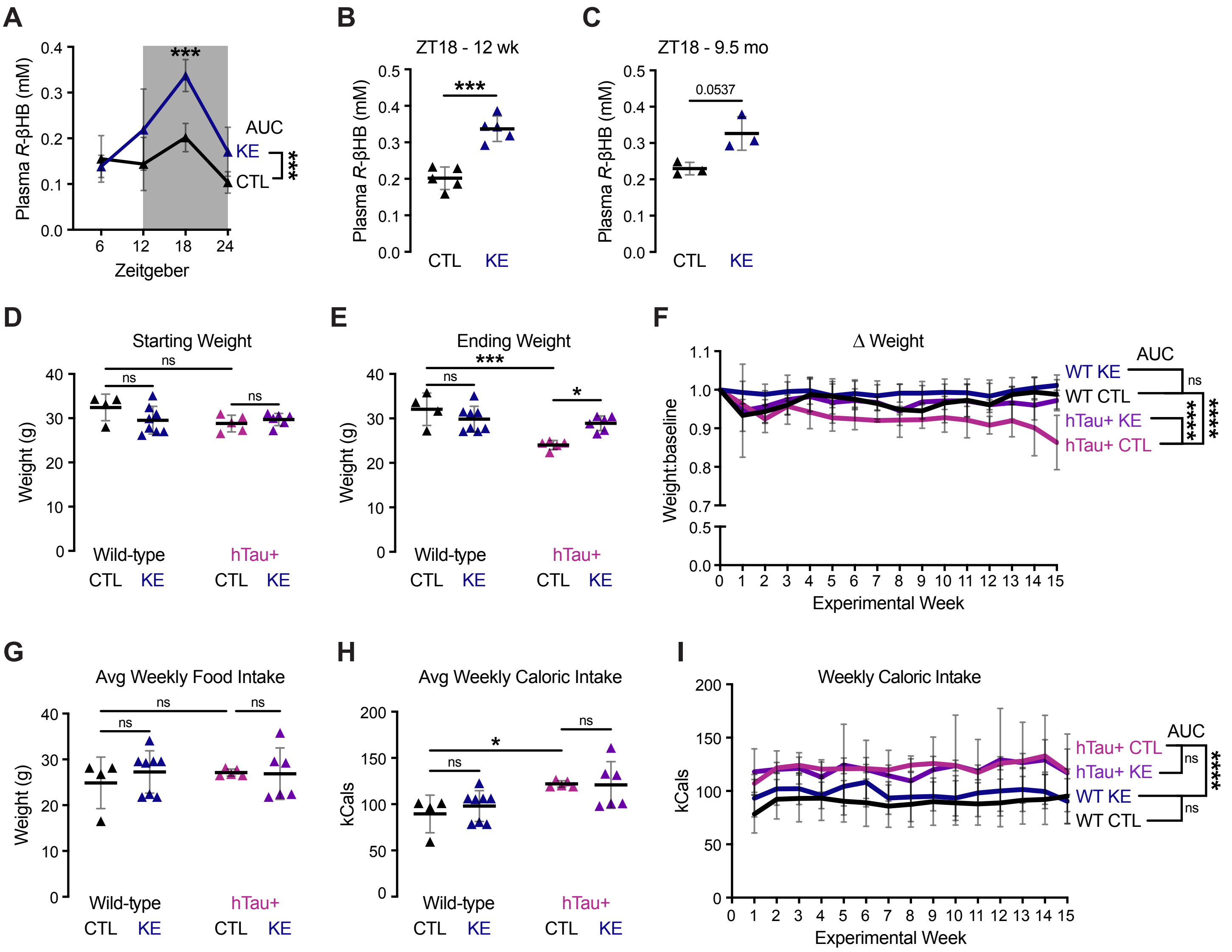

bioRxiv preprint doi: https://doi.org/10.64898/2026.01.30.702936; this version posted February 2, 2026. The copyright holder for this preprint (which was not certified by peer review) is the author/funder, who has granted bioRxiv a license to display the preprint in perpetuity. It is made available under aCC-BY 4.0 International license.

Figure S2, related to Figure 2: Ketone ester diet minimally changes highly soluble (RAB and PBS) species of Tau.

(A) Representative Western blot showing RAB-soluble cortical fractions from WT and hTau+ mice fed with CTL or KE diets. Arrows indicate p-hTau band quantified for AT8 and PHF1. hTau, human Tau; msTau, mouse Tau.

(B-G) Quantifications of p-hTau (B-D), hTau (E-F), and total Tau (G) in RAB-soluble cortical fractions.

(H) Representative Western blot for Tau species in PBS-soluble cortical lysates from WT and hTau+ mice fed with CTL or KE diets. Lysates were used for a Tau seeding assay in HEK293T biosensor cells (**Fig. 2N**).

(I-K) Quantifications of p-hTau (I-J) and total hTau (K) from PBS-soluble cortical lysates.

Data are represented as mean ± SD. Each point represents one mouse. Data were normalized to the hTau+ CTL group. * $p < 0.05$, ns not significant by Welch's t-test.

bioRxiv preprint doi: https://doi.org/10.64898/2026.01.30.702936; this version posted February 2, 2026. The copyright holder for this preprint (which was not certified by peer review) is the author/funder, who has granted bioRxiv a license to display the preprint in perpetuity. It is made available under aCC-BY 4.0 International license.

Figure S2

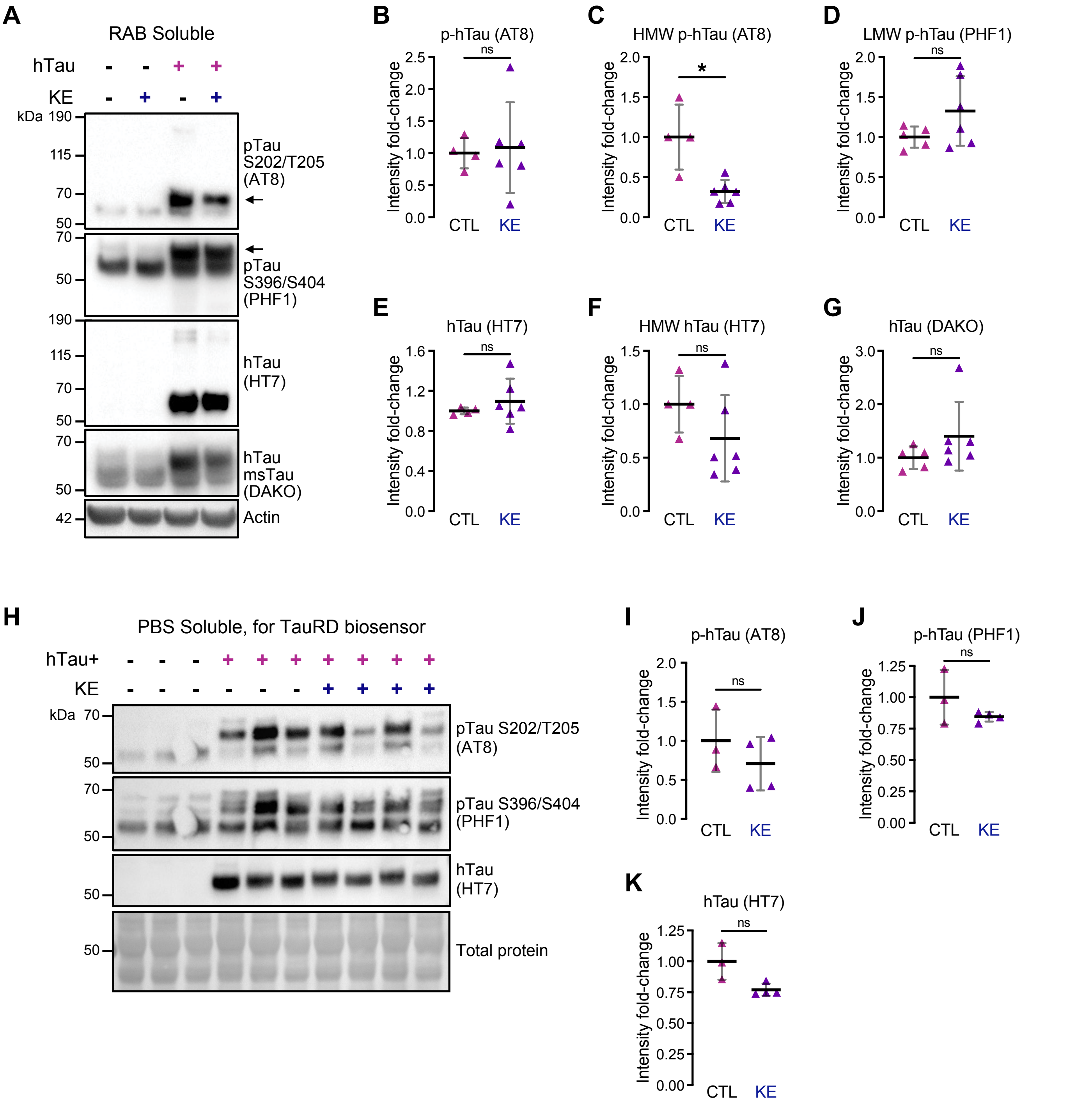

bioRxiv preprint doi: https://doi.org/10.64898/2026.01.30.702936; this version posted February 2, 2026. The copyright holder for this preprint (which was not certified by peer review) is the author/funder, who has granted bioRxiv a license to display the preprint in perpetuity. It is made available under aCC-BY 4.0 International license.

Figure S3, related to Figure 3: Hippocampal snRNA sequencing of KE diet-fed PS19 mice identifies 14 distinct cell types, with the KE diet reverting global cell-type distribution and metabolic profiles of hTau+ mice to WT levels.

(A) Relative expression of canonical cell type markers in hippocampal snRNA seq experiment within diet study. 14 distinct clusters were identified. Circle saturation represents the normalized average expression of marker genes of all cells within a cluster, and size represents percent of cells within a cluster expressing a given marker gene. ExcN, excitatory neurons; InhN, inhibitory neurons; Micros, microglia; Astros, astrocytes; Oligos, oligodendrocytes; OPCs, oligodendrocyte precursor cells; and Vasc, vasculature-associated cells.

(B-C) Cell- and mouse-level relative expression of *Gfap* (B), a reactive astrocytic marker, in the astrocyte cluster. The KE diet ameliorates elevated expression levels and proportion of astrocytes per mouse expressing *Gfap* (C) in hTau+ mice.

(D-E) Cell- and mouse-level relative expression of *Clec7a* (D), a reactive microglial marker, in the microglia cluster. The KE diet ameliorates elevated expression levels and proportion of microglia per mouse expressing *Clec7a* (E) in hTau+ mice.

(F-G) Relative expression levels of *Oxct1* (F) and *Slc16a7* (MCT2) (G), genes involved in ketone body utilization, quantified at the single-cell level by ExcN cluster.

(H) Violin plot representing net (positive - negative) reaction scores for Bdh1-dependent ketolysis at the micropooled cell level by ExcN cluster. A larger net reaction score indicates more positive flux in the indicated reaction direction. [m] mitochondria.

(I-J) Volcano plots of binary comparisons between hTau+ CTL v. WT CTL (I) or v. hTau+ KE (J) on micropooled ExcN1 net reaction scores for butanoate subsystem metabolites. Colored dots were significant by adjusted $p < 0.05$. [m] mitochondria, [c] cytosol.

(K-L) Principal component (PC) analysis of Compass results for all ExcNs for sugar (K) and lipid (L) metabolism pathways. A single dot represents 100 micropooled cells within the same ExcN cluster and mouse.

Data at the mouse level are represented as mean ± SD. ** $p < 0.01$, *** $p < 0.001$, **** $p < 0.0001$ relative to WT CTL, and # $p < 0.05$, ## $p < 0.01$, ### $p < 0.001$, #### $p < 0.0001$ relative to hTau+ CTL, unless comparisons are specified, by DESeq2 test (B, D, F-G), Šídák's multiple comparisons test (C, E), and Welch's t-test with Bonferroni's correction (H-J).

bioRxiv preprint doi: https://doi.org/10.64898/2026.01.30.702936; this version posted February 2, 2026. The copyright holder for this preprint (which was not certified by peer review) is the author/funder, who has granted bioRxiv a license to display the preprint in perpetuity. It is made available under aCC-BY 4.0 International license.

Figure S3

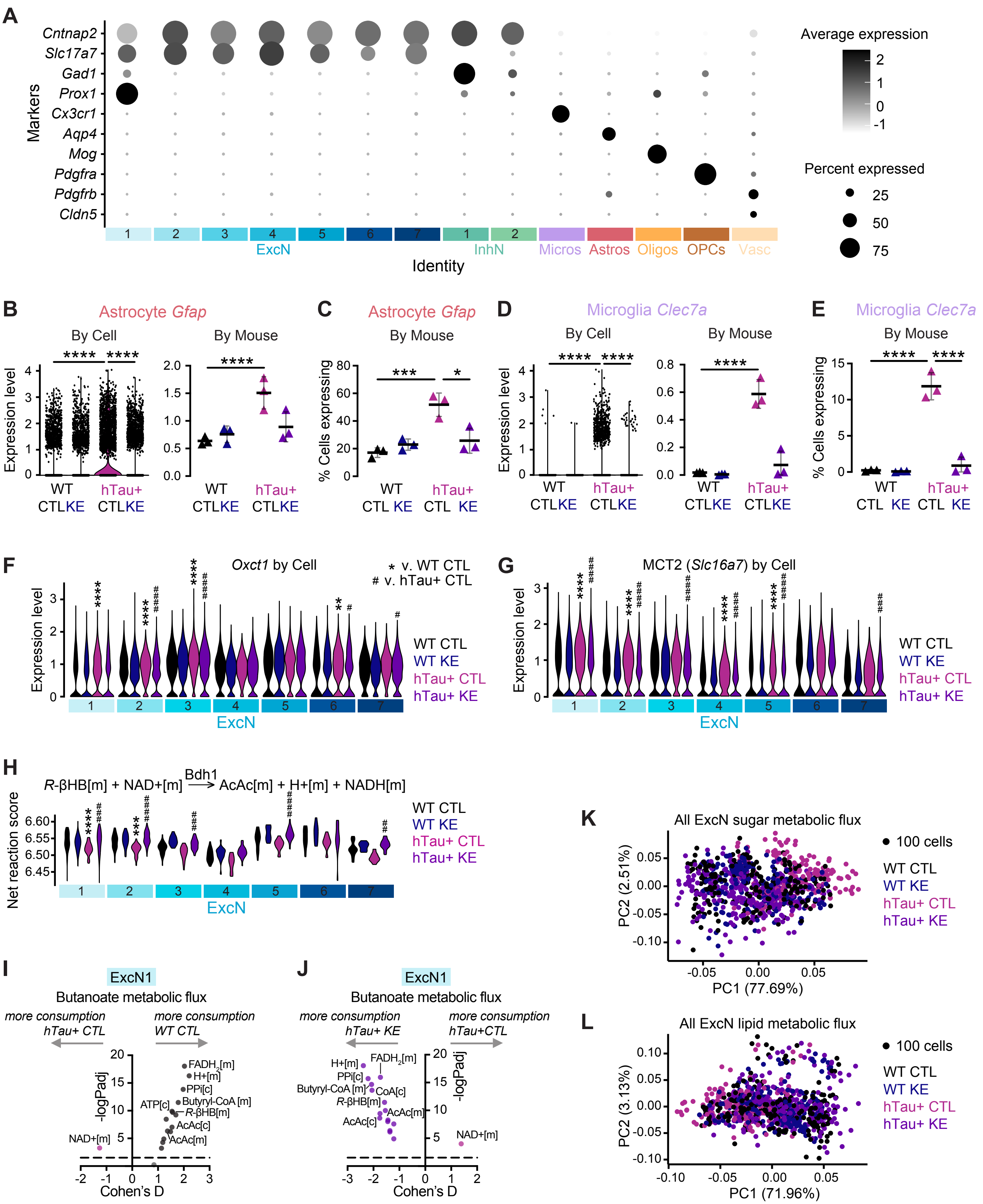

bioRxiv preprint doi: https://doi.org/10.64898/2026.01.30.702936; this version posted February 2, 2026. The copyright holder for this preprint (which was not certified by peer review) is the author/funder, who has granted bioRxiv a license to display the preprint in perpetuity. It is made available under aCC-BY 4.0 International license.

Figure S4, related to Figure 3: Analysis of ExcN clusters illustrates KE diet reversal to WT levels of synaptic and proteostatic status of ExcNs in hTau+ mice.
(A-H) Relative expression levels of *α7 nAChR* (A-B), *Hspa5* (BiP) (C-D), *Eif2ak3* (PERK) (E-F), and *Hsp90ab1* (G-H) represented both at the single-cell level by ExcN cluster (A, C, E, G) and at the mouse level by pseudobulking (B, D, F, H).
(I) Percentage of total ExcNs expressing synaptic markers *C1qa*, *C1qb*, and *C1qc*, quantified at the mouse level.
(J) Percentage of total microglia expressing phagocytosis marker *Lgals3* (Gal-3), quantified at the mouse level.
Data at the mouse level are represented as mean ± SD. * $p < 0.05$, ** $p < 0.01$, *** $p < 0.001$, **** $p < 0.0001$ relative to WT CTL, and # $p < 0.05$, ## $p < 0.01$, #### $p < 0.0001$ relative to hTau+ CTL, unless comparisons are specified. ns not significant. DESeq2 test was used for expression level comparisons (A-H) and Šídák's multiple comparisons test for percent expression comparisons (I-J).

bioRxiv preprint doi: https://doi.org/10.64898/2026.01.30.702936; this version posted February 2, 2026. The copyright holder for this preprint (which was not certified by peer review) is the author/funder, who has granted bioRxiv a license to display the preprint in perpetuity. It is made available under aCC-BY 4.0 International license.

Figure S4

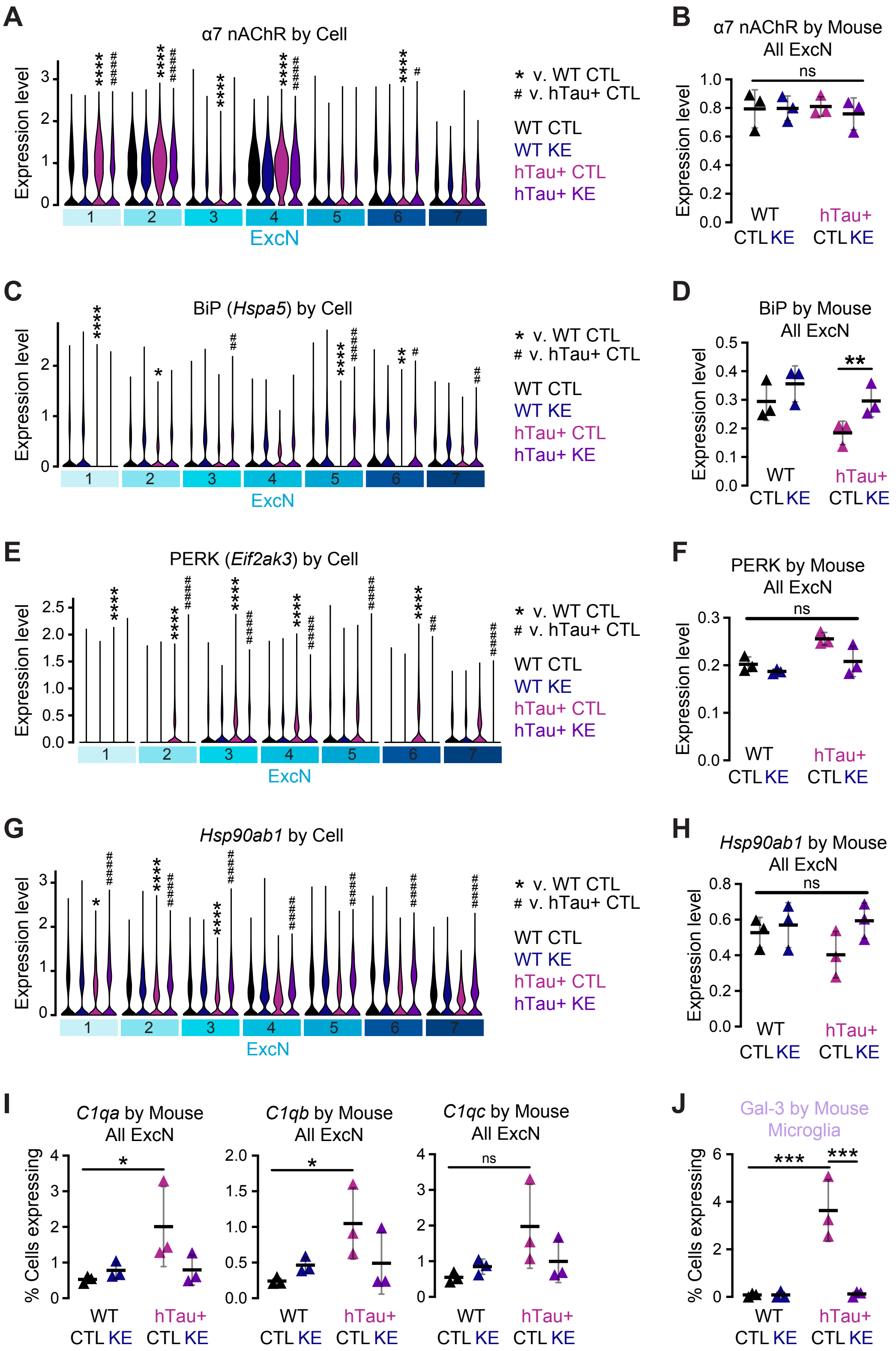

bioRxiv preprint doi: https://doi.org/10.64898/2026.01.30.702936; this version posted February 2, 2026. The copyright holder for this preprint (which was not certified by peer review) is the author/funder, who has granted bioRxiv a license to display the preprint in perpetuity. It is made available under aCC-BY 4.0 International license.

Figure S5, related to Figure 5: βHB alters neuronal Tau interactome, strengthening Tau's interaction with endomembrane and synaptic systems.
(A) Cartoon showing subcellular location of manually curated synaptic system proteins whose interactions with Tau are strengthened with βHB. The color of the protein signifies the enantiomer of βHB that is significant. Significance cut-offs were defined as $p < 0.05$ and $|FC| > 0.5$. The "+" indicates that the change in interaction was significant in one enantiomer of βHB while the other enantiomer had a p-value < 0.1.
(B-F) Normalized protein abundances of Tau interactors SNAP91 (B), STAM (C), HGS (D), TSG101 (E), and ALYREF (F) with βHB treatments.
P-values and FCs were determined by linear regression model to remove variance shared with APEX-Tub, representing artifactual interaction changes due to changes in the background proteome (see **Methods**). Data are represented as mean ± SD. * $p < 0.05$, ** $p < 0.01$, *** $p < 0.001$, **** $p < 0.0001$ by linear regression model.

bioRxiv preprint doi: https://doi.org/10.64898/2026.01.30.702936; this version posted February 2, 2026. The copyright holder for this preprint (which was not certified by peer review) is the author/funder, who has granted bioRxiv a license to display the preprint in perpetuity. It is made available under aCC-BY 4.0 International license.

Figure S5

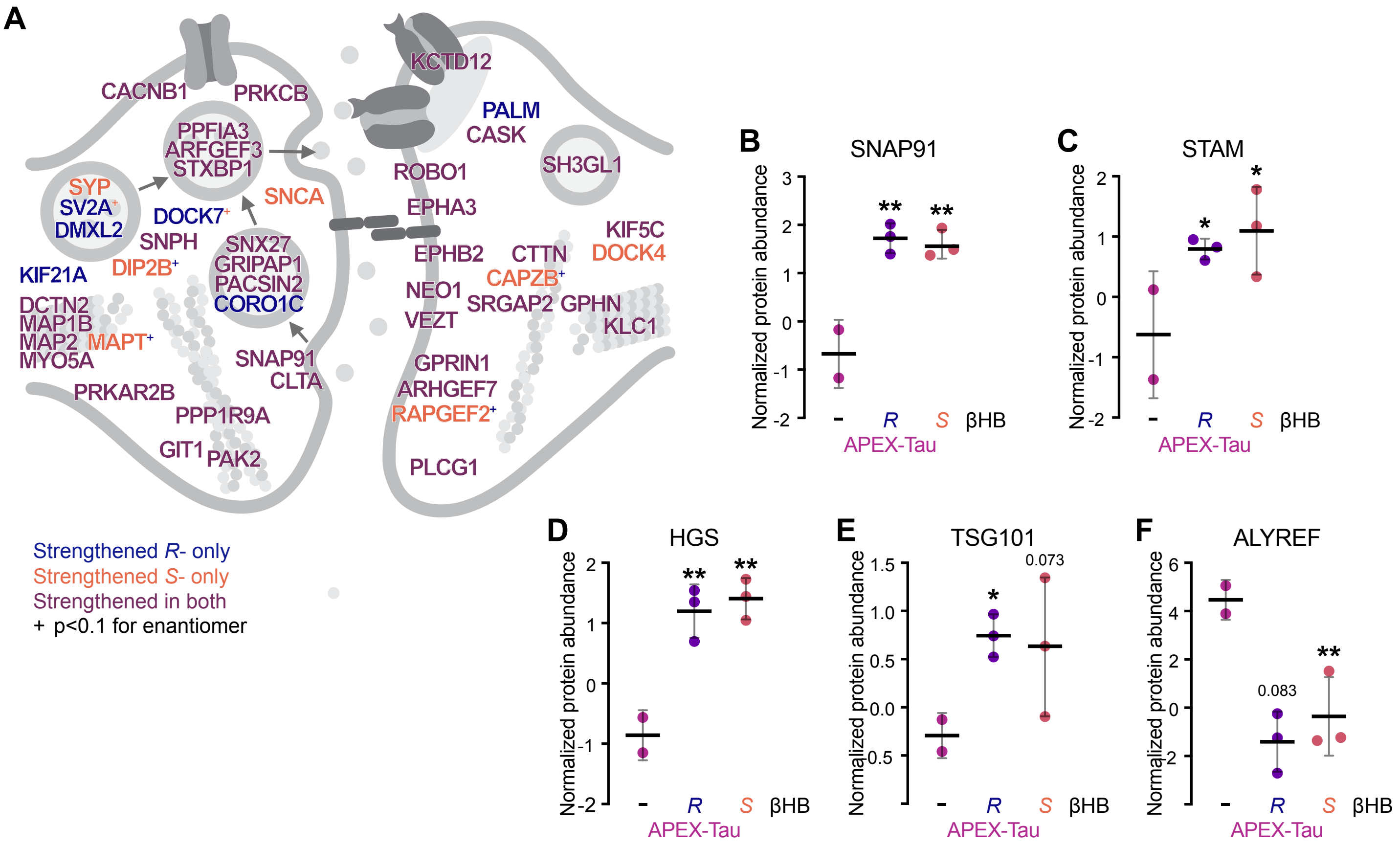

bioRxiv preprint doi: https://doi.org/10.64898/2026.01.30.702936; this version posted February 2, 2026. The copyright holder for this preprint (which was not certified by peer review) is the author/funder, who has granted bioRxiv a license to display the preprint in perpetuity. It is made available under aCC-BY 4.0 International license.

Figure S6: βHB enhances *Ubb* expression and increases autophagic vesicles in neurons; OPTN knockdown in neurons.

(A) Relative expression levels of *Ubb* at the single-cell level by ExcN cluster. * $p < 0.05$, *** $p < 0.001$, and **** $p < 0.0001$ relative to WT CTL, #### $p < 0.0001$ relative to hTau+ CTL. *Ubb*, ubiquitin.

(B) Quantification of total autophagic vesicles in primary neurons, corresponding to **Fig. 6F-H** (autophagosomes + autolysosomes). Each point represents an individual Map2+ neuron, with cells in the same well stacked into one column. Thick, color-coded bars represent the well mean (n = 8-15 cells/well), and black bars represent the overall group mean ± SD (n = 3 wells/group, from separate batches). * $p < 0.05$, ** $p < 0.01$ by linear mixed-effects model with Tukey *post-hoc* test.

(C-D) Representative Western blot (B) and quantification (C) of OPTN in primary neurons, corresponding to **Fig. 6I**. Each point represents one independent well, normalized to the average of the NaCl-treated values for its respective plate. **** $p < 0.0001$ by Welch's two-way ANOVA.

(E) Quantification of relative intracellular Tau levels in primary neurons, corresponding to **Fig. 6I**. ns not significant by Šídák's multiple comparisons test.

Data for D-E are represented as mean ± SD.

bioRxiv preprint doi: https://doi.org/10.64898/2026.01.30.702936; this version posted February 2, 2026. The copyright holder for this preprint (which was not certified by peer review) is the author/funder, who has granted bioRxiv a license to display the preprint in perpetuity. It is made available under aCC-BY 4.0 International license.

Figure S6

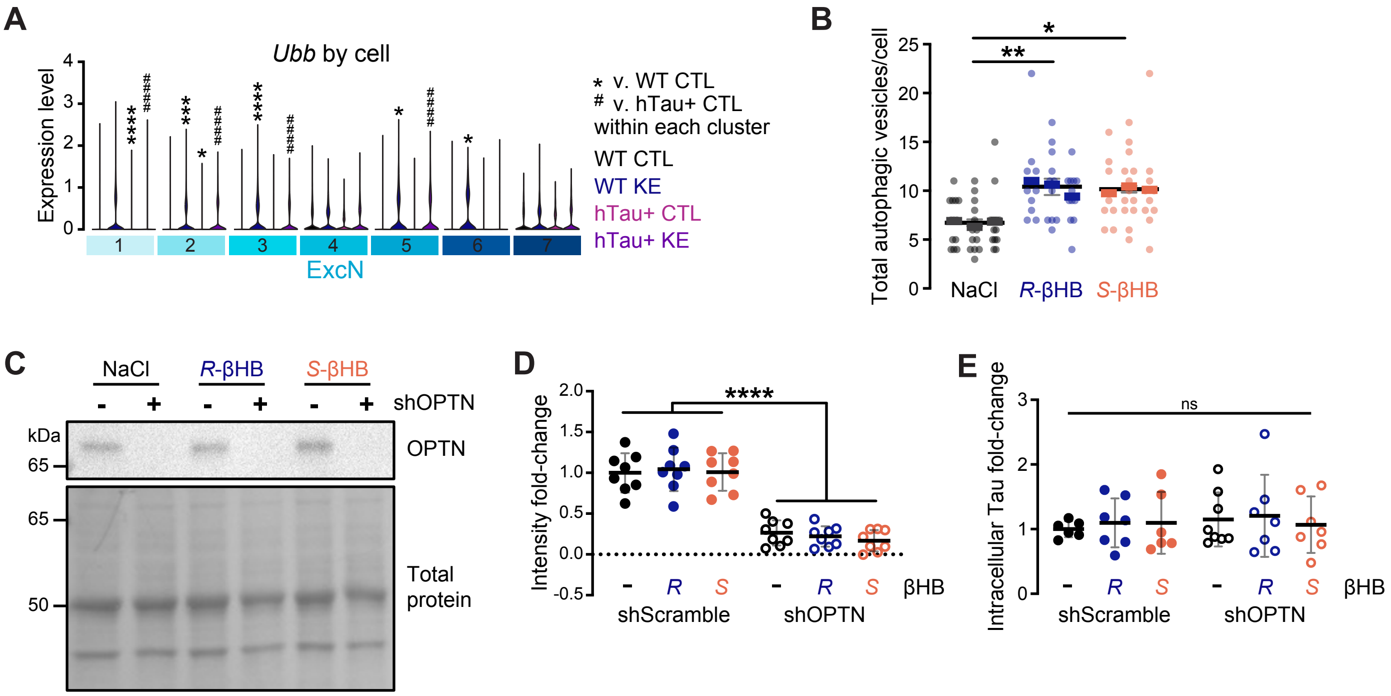

Supplement: Supplement 1 [file NIHPP2026.01.30.702936v1-supplement-1.pdf]
